# YKL-40 is highly expressed in the epicardial adipose tissue of patients with atrial fibrillation and associated with atrial fibrosis

**DOI:** 10.1186/s12967-018-1598-0

**Published:** 2018-08-15

**Authors:** Qing Wang, Hua Shen, Jie Min, Yang Gao, Kai Liu, Wang Xi, Jie Yang, Liang Yin, Jibin Xu, Jian Xiao, Zhinong Wang

**Affiliations:** Center for Comprehensive Treatment of Atrial Fibrillation, Department of Cardiothoracic Surgery, Changzheng Hospital, Second Military Medical University, Shanghai, 200003 China

**Keywords:** YKL-40, Epicardial adipose tissue, Atrial fibrosis, Atrial fibrillation, Body mass index

## Abstract

**Background:**

YKL-40 (*CHI3L1*) is a novel biomarker for inflammation, tissue remodeling, and fibrosis, as well as cardiovascular diseases. We investigated the association between YKL-40 expression in epicardial adipose tissue (EAT) and atrial fibrosis in patients with atrial fibrillation (AF).

**Methods:**

Blood samples, subcutaneous adipose tissue (SAT), paracardial adipose tissue (PAT), EAT, and adjacent atrial myocardium were acquired from patients receiving coronary artery bypass grafts. The patients were divided into the AF group (n = 28) and the sinus rhythm (SR) group (n = 36).

**Results:**

We did not detect a significant difference in the serum YKL-40 levels in the SR and AF groups (*P *= 0.145). Quantitative real-time PCR showed that YKL-40 (*CHI3L1*) mRNA levels in the EAT were significantly higher than in the SAT or PAT of AF patients, or the EAT of SR patients (All *P *< 0.001). We found similar results for YKL-40 protein levels by immunohistochemistry. Masson staining showed significantly more fibrosis in AF patients than in SR patients (*P *< 0.001). Western blotting indicated that AF patients had significantly higher expression of collagen I (*P *= 0.039). We found a linear relationship between YKL-40 mRNA expression and the collagen volume fraction of the atrial myocardium (y = 3.576x + 26.205, *P *< 0.001). Multivariate linear regression analysis revealed that body mass index is an independent risk factor for YKL-40 expression in EAT (β = 0.328, *P *= 0.011).

**Conclusions:**

YKL-40, which is highly expressed in the EAT of patients with AF, is affected by body mass index and associated with atrial fibrosis, which may contribute to the development of AF.

## Background

Atrial fibrillation (AF), the commonest sustained arrhythmia in clinics, burdens millions of people worldwide, with increasing prevalence, mortality, and morbidity [1]. As a result, it has become a hotspot in cardiovascular research, both in clinical and basic research settings. However, its underlying mechanism is still elusive. Atrial remodeling plays a key role in the pathogenesis of AF [2]. Atrial fibrosis, the fundamental process of atrial structural remodeling, greatly contributes to the incidence and maintenance of AF [3].

Epicardial adipose tissue (EAT), once merely thought to be a protective and metabolic tissue for the heart, has recently been found to be related to several cardiovascular diseases, especially AF [4]. Nevertheless, the specific relationship between EAT and AF remains elusive. YKL-40 (CHI3L1), an emerging biomarker in cardiovascular disease, is named for its first 3 terminal amino acids [tyrosine (Y), lysine (K), and leucine (L)] and its apparent molecular weight [5]. YKL-40 is expressed by the gene chitinase-3-like protein 1 (*CHI3L1*) in several cell types, such as vascular smooth muscle cells, monocytes, and macrophages [6]. YKL-40 was first described as an inflammatory glycoprotein, which involved in asthma and lung function [7]. Recent research showed that it can be highly expressed in visceral adipose tissue [8] and participate in cardiovascular disease and diabetes [5]. In this study, we investigated YKL-40 expression in EAT and its relationship with atrial fibrosis in AF patients.

## Methods

### Study population

From March 2016 to Dec 2017, a total of 64 patients who received coronary artery bypass grafts in Changzheng Hospital affiliated with the Second Military Medical University, were enrolled in this study: 36 patients with sinus rhythm (SR) and 28 patients with persistent or permanent AF. Exclusion criteria included structural heart disease, severe hepatic or renal dysfunction, metabolic disease, infectious disease, cancer, or age over 80 years. AF was determined by 12-lead electrocardiogram. The baseline characteristics of all patients were collected for analysis.

### Sample acquisition

Peripheral venous blood samples were acquired in the first morning after the admission. The serum was centrifuged and stored at − 80 °C. Adipose tissue was obtained during surgery, before the cardiopulmonary bypass. The different types of adipose tissue (average 0.5 g each) were collected from each patient, including subcutaneous adipose tissue (SAT) at the chest incision, paracardial adipose tissue (PAT) from the pericardium, and EAT from the atrioventricular groove next to the right atrial appendage. Each biopsy was divided into two portions; one was frozen immediately at − 80 °C for RNA isolation, and the other was immersed in neutralized formalin for immunohistochemistry. The adjacent atrial myocardium (average 0.1 g) from the right atrial appendage tissue was also collected and likewise divided into two portions.

### Enzyme-linked immunosorbent assay

A commercial YKL-40 two-site, sandwich-type enzyme-linked immunosorbent assay kit (Quidel Corporation, San Diego, CA, USA) was used to measure serum YKL-40 levels.

### RNA isolation and quantitative real-time PCR

TRIzol^®^ reagent (Invitrogen, Carlsbad, CA, USA) was used to extract total RNA; the purity of the isolated RNA was assessed by measurement of the optical density at 260 nm and 280 nm. Reverse transcription was performed using a High-Capacity cDNA Reverse Transcription Kit (Applied Biosystems, Foster City, CA, USA), according to the manufacturer’s instructions. SYBR^®^ Premix Ex Taq™ (TakaRA Bio, Tokyo, Japan) was used to prepare the quantitative real-time PCR samples. An ABI Prism 7900 Detector System (Applied Biosystems) was used to perform the quantitative real-time PCR. The primers were designed using Primer Premier 6.0 software (Premier Biosoft, Palo Alto, CA, USA). The primer sequences used in this study were as follows: YKL-40 (*CHI3L1*), forward 5′-GTGAAGGCGTCTCAAACAGG-3′, reverse 5′-GAAGCGGTCAAGGGCATCT-3′; collagen I (*COL1A1*), forward 5′-CCAAGACGAAGACATCCCACC-3′, reverse 5′-CAGTTGTCGCAGACGCAGAT-3′; collagen III (*COL3A1*), forward 5′-TCGCTCTGCTTCATCCCACTAT-3′, reverse 5′-CTTCCAGACATCTCTATCCGCAT-3′; β-actin (*ACTB*), forward 5′-CATGTACGTTGCTATCCAGGC-3′, reverse 5′-CTCCTTAATGTCACGCACGAT-3′. Relative gene expression was calculated using the threshold cycle value (C_T_) and the formula 2^−ΔΔCT^.

### Immunohistochemistry

The paraffin-embedded sections were placed at 60 °C for 1 h, then dewaxed, rehydrated, and rinsed 2–3 times with PBS, each time for 3 min. Next, the sections were subjected to 3% hydrogen peroxide solution at room temperature for 8 min, then rinsed 3 times with PBS (pH 7.2–7.6), each time for 5 min. Sections were incubated with primary antibodies (CHI3L, Abcam, Cambridge, UK) overnight at 4 °C in a moist chamber, then rinsed 3 times with PBS (pH 7.2–7.6). Then, the sections were incubated with secondary antibodies for 1 h at 4 °C, then rinsed 3 times with PBS; 50–100 μl DAB reagent was added. The slices were observed under a microscope. The integrated optical density (IOD) of positively stained tissue was calculated to quantify the expression of YKL-40. The IOD of each tissue section was calculated from eight different 400× magnified fields.

### Masson staining

The samples were dewaxed conventionally to water, then washed thoroughly with water. We stained with the hematoxylin from the Masson staining kit for 5 min, then differentiated the sections with ethanolic hydrochloric acid, and rinsed them thoroughly with water. The sections were then rinsed to blue-black with flowing water; the vermic acid red wine was used to stain for 3–8 min, then sections were rinsed with distilled water, and differentiated with 1% phosphomolybdic acid for 2 min. We stained with the aniline blue complex for 3 min, then differentiated the slides with 1% glacial acetic acid for 1–2 min. The samples were sliced, dehydrated, rendered transparent with xylene, and sealed with optical rubber. The sections were observed under a light microscope, and the image data were collected to calculate the volume fraction of collagen (CVF% = average collagen area/area of total field × 100).

### Western blotting

The atrial myocardium samples were weighed and treated with cold Radio-Immunoprecipitation Assay (RIPA) lysis buffer (1 ml/100 mg). The homogenized samples were centrifuged (12,000 rpm, 10 min, 4 °C) and a BCA protein assay kit (Yeason Biotech, Shanghai, China) was used to determine their protein concentrations. Loading buffer was added to diluted supernatants to adjust concentrations and volumes. Afterward, samples with equal protein content were subjected to sodium dodecyl sulfate–polyacrylamide gel electrophoresis on a 10% polyacrylamide gel, then transferred to a polyvinylidene difluoride membrane (Millipore, Boston, MA, USA). Next, the samples were blocked with blocking buffer containing 5% bovine serum albumin for 1 h at room temperature, followed by incubation with antibodies including anti-collagen I (Boster Biological Technology, Wuhan, China) and anti-collagen III (Boster Biological Technology, Wuhan, China), overnight at 4 °C, with anti-GAPDH used as an internal control. The membranes were rinsed 3 times for 10 min with TBST, followed by incubation with the secondary antibody for 1 h. The membranes were washed 3 times with TBST for 15 min. Then, the membranes were developed with chemiluminescence solution A and B mixed at a 1:1 ratio, along with the developing substrate. Image J software was used to calculate the relative optical density of the bands.

### Statistical analysis

Continuous data, when distributed normally, were expressed as mean ± standard deviation, while the categorical data were expressed as percentages. Abnormally distributed variables were described as median and interquartile range (IQR). The baseline characteristics of the two groups were compared with Student’s t-test and the Chi square test. Immunohistochemistry IOD and mRNA levels between groups were compared using Mann–Whitney test, while the differences among the three types of adipose tissue were detected by Kruskal–Wallis test. The CVF% calculated from the Masson staining and the relative optical density of western blotting between two groups were compared with Student’s t-test. A correlation analysis between mRNA expression and CVF% was conducted by univariate linear regression. A multivariate linear regression model was used to analyze the risk factors affecting the expression of YKL-40 in EAT, and a enter pattern was adopted for building the model. All data were analyzed with SPSS 22.0 software (IBM, Almonte, NY, USA). Differences were considered significant when *P *< 0.05.

## Results

### Patient characteristics

**Fig. 1:**
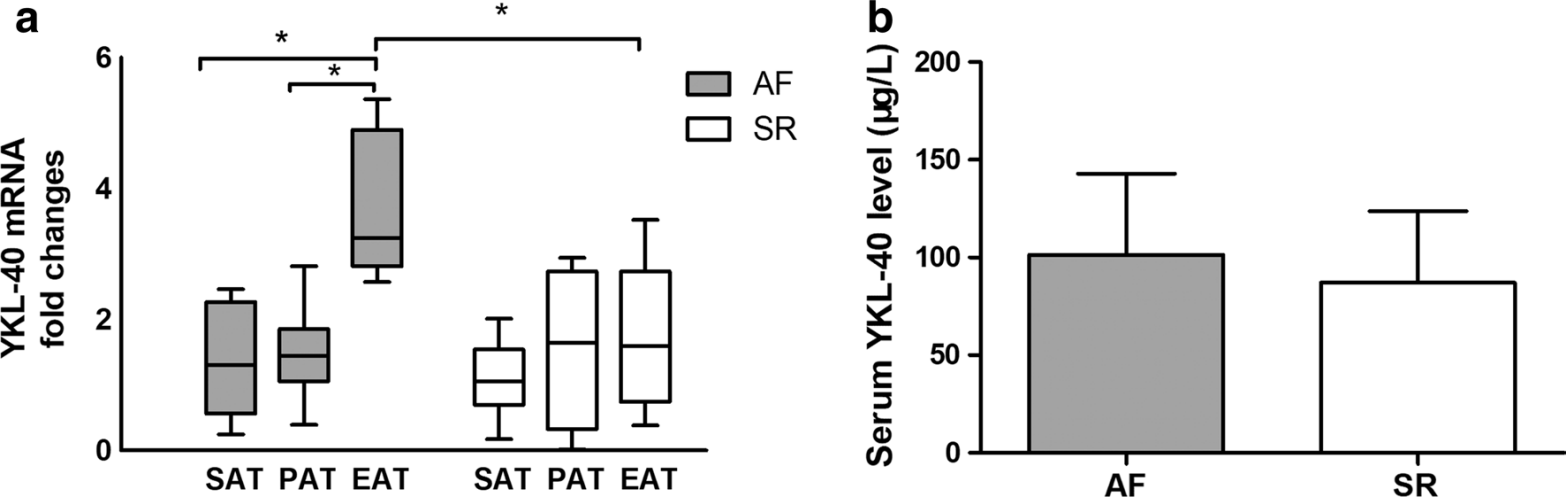
Quantitative real-time PCR results of YKL-40 (*CHI3L1*) expression in the adipose tissue and ELISA results of serum YKL-40 level for patients with atrial fibrillation (AF, n = 28) and sinus rhythm (SR, n = 36). **a** For AF patients, the YKL-40 expression was significantly higher in EAT than SAT and PAT. Between groups, YKL-40 in EAT of AF patients was significantly higher than that of SR patients. **b** No significant difference in serum YKL-40 between AF patients and SR patients. *P < 0.001. *SAT* subcutaneous adipose tissue, *PAT* paracardial adipose tissue, *EAT* epicardial adipose tissue

Patient baseline characteristics are shown in Table 1. The demographic data were similar in the two groups including age, gender, body mass index (BMI), and smoking status (*P *> 0.05). In addition, there were no cardiac function disparities between the two groups, as manifested in NYHA class or left ventricular ejection fraction (LVEF) (*P *> 0.05). The patients in both groups had similar proportions of comorbidities, including hypertension, type 2 diabetes mellitus, stroke, and chronic obstructive pulmonary disease (COPD). However, the average left atrial diameter (LAD) of the AF group was significantly larger than that of the SR group (t = 4.547, *P *< 0.001). Showed in Fig. 1b, We also tested serum YKL-40, which was higher in the AF group, but the difference was not statistically significant (t = 1.475, *P *= 0.145).

**Table 1:** Baseline characteristics of patients in the atrial fibrillation (AF) and sinus rhythm (SR) groups

Variables	SR group (n = 36)	AF group (n = 28)	χ^2^/t	*P*
Demographics				
Age (year)	67.8 ± 8.8	66.5 ± 8.1	0.605	0.547
Gender (percent male)	20 (55.6%)	16 (57.1%)	0.016	0.899
BMI (kg/m^2^)	23.8 ± 2.9	25.6 ± 3.6	1.652	0.104
Smoking	10 (27.8%)	8 (28.6%)	0.005	0.944
NYHA functional class			2.709	0.439
I	5 (13.9%)	3 (10.7%)		
II	12 (33.3%)	12 (42.9%)		
III	16 (44.4%)	8 (28.6%)		
IV	3 (8.3%)	5 (17.9%)		
Echocardiography				
LVEF (%)	58.8 ± 4.4	59.3 ± 5.0	0.391	0.697
LAD (mm)	37.9 ± 5.8	43.8 ± 3.9	4.547	< 0.001
Comorbidities				
Hypertension	10 (27.8%)	6 (21.4%)	0.339	0.561
T2DM	12 (33.3%)	8 (28.6%)	0.166	0.683
Stroke	2 (5.6%)	4 (14.3%)	1.413	0.235
COPD	4 (11.1%)	4 (14.3%)	0.145	0.703
Serum YKL-40 (μg/l)	87.0 ± 36.6	101.5 ± 41.5	1.475	0.145

*BMI* body mass index, *LVEF* left ventricular ejection fraction, *LAD* left atrial diameter, *T2DM* type 2 diabetes mellitus, *COPD* chronic obstructive pulmonary disease, *NYHA* New York Heart Association

### YKL-40 expression

YKL-40 mRNA expression in the adipose tissue of the two groups is shown in Fig. 1a. In the SR group, we detected no significant differences among the three types of adipose tissue (χ^2^ = 5.686, *P *= 0.058). In the AF group, YKL-40 mRNA expression differed significantly among the types of adipose tissue (χ^2^ = 52.531, *P *< 0.001); it was significantly more highly expressed in EAT than in SAT or PAT (EAT vs SAT: 3.25 (2.09) vs 1.30 (1.70), *P *< 0.001; EAT vs PAT: 3.25 (2.09) vs 1.45 (0.80), *P *< 0.001). YKL-40 mRNA expression in SAT and PAT did not differ between the groups (SAT: *P *= 0.256; PAT: *P *= 0.735). However, it was significantly more highly expressed in the EAT of the AF group than the SR group (*P *< 0.001).

**Fig. 2:**
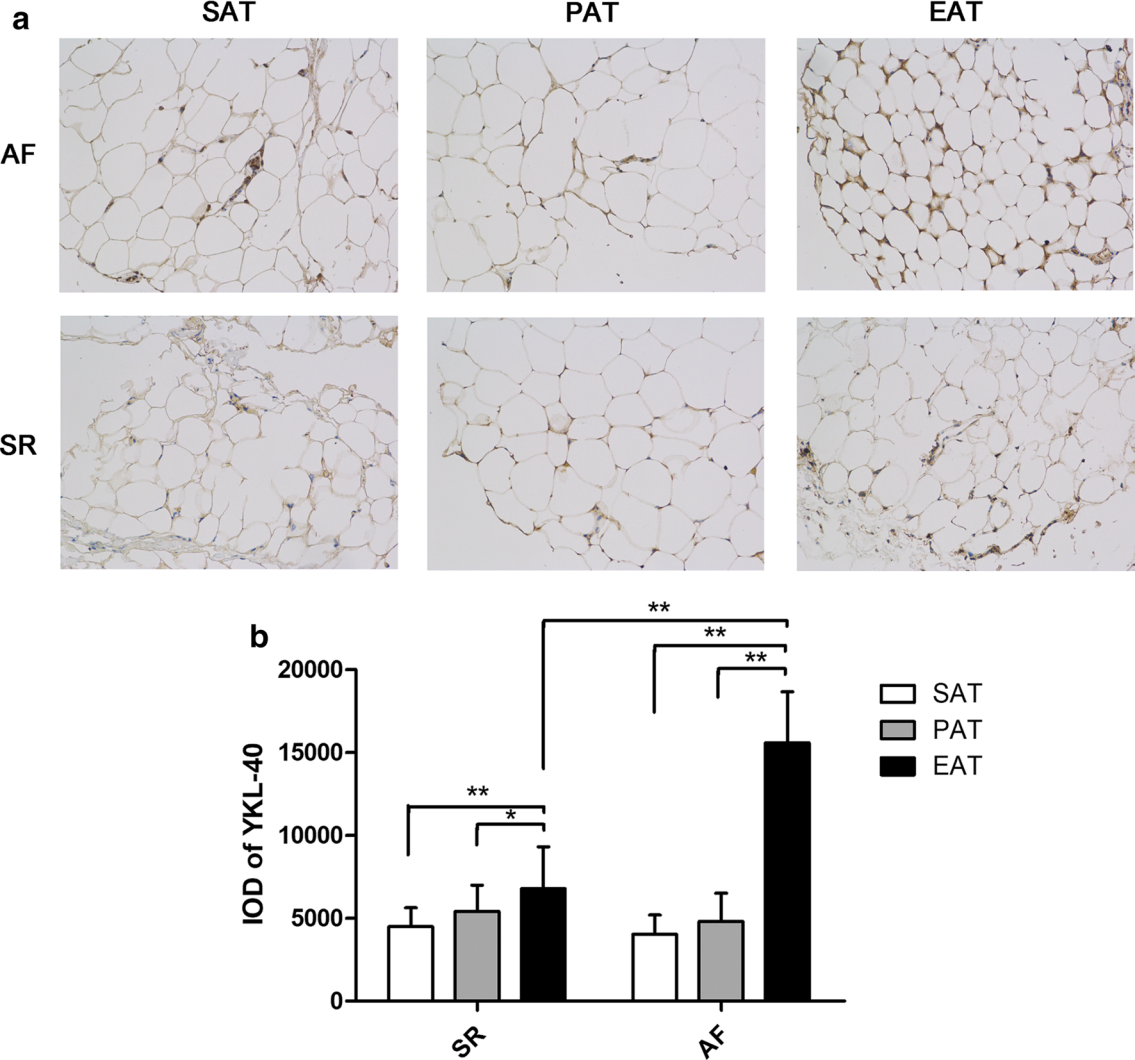
Immunohistochemical integrated optical density analysis of YKL-40 expression in the adipose tissue of patients with atrial fibrillation (AF, n = 28) and sinus rhythm (SR, n = 36). **a** Representative sections of three types of adipose tissue in the AF and SR groups. The EAT of AF group was obviously more evident in staining. **b** Quantitative results of IOD in sections of three types of adipose tissue in the AF and SR groups. *P = 0.007, **P < 0.001. *SAT* subcutaneous adipose tissue, *PAT* paracardial adipose tissue, *EAT* epicardial adipose tissue

As demonstrated in Fig. 2a, the representative EAT sections from the AF group stained more deeply than the other types of adipose tissue, including the adipose tissue of the SR group. As illustrated in Fig. 2b, the quantitative IOD results from the immunohistochemistry sections corresponded with the mRNA expression results. In the SR group, we found significant differences among the three types of adipose tissue (F = 13.814, *P *< 0.001); the IOD of the EAT sections was significantly higher than that of the SAT sections and PAT sections (EAT vs SAT: 6767 ± 2548 vs 4486 ± 1135, *P *< 0.001; EAT vs PAT: 6767 ± 2548 vs 5410 ± 1583, *P *= 0.007). The differences in the IOD among the three types of adipose tissue in the AF group were significant (F = 255.311, *P *< 0.001). The IOD of the EAT sections was higher than that of both the SAT and PAT sections (EAT vs SAT: 15,582 ± 3080 vs 4808 ± 1693, *P *< 0.001; EAT vs PAT: 15,582 ± 3080 vs 4032 ± 1166, *P *< 0.001). Between groups, the IOD of the EAT sections was higher in the AF group than in the SR group (t = 12.528, *P *< 0.001), although it did not differ in the SAT (t = 1.569, *P *= 0.122) and PAT sections (t = 1.466, *P *= 0.148).

### Atrial fibrosis

**Fig. 3:**
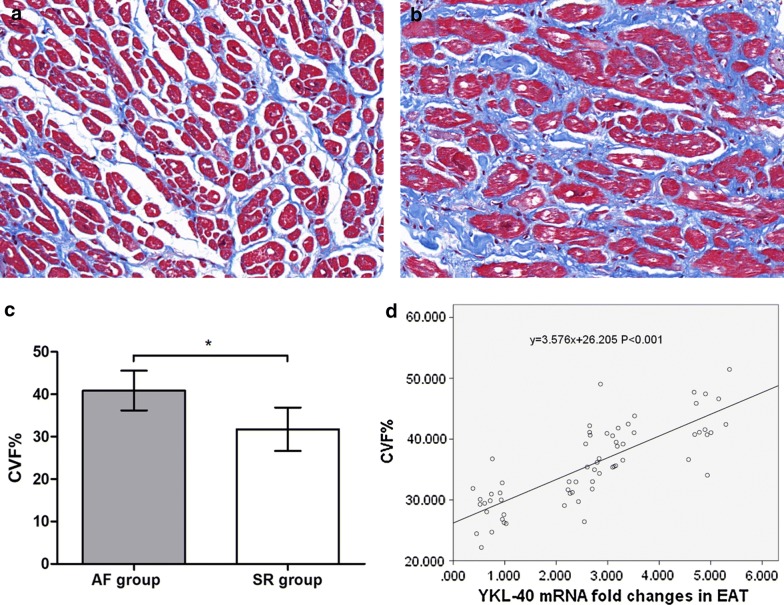
Masson staining and quantitative results of atrial myocardium from patients with atrial fibrillation (AF, n = 28) and sinus rhythm (SR, n = 36). **a** Masson staining of representative sections from the SR group. All cardiomyocytes were regularly arrayed with little interstitial fibers. **b** Masson staining of representative sections from the AF group. The cardiomyocytes were disrupted by increased interstitial fibers. **c** Quantitative results of Masson staining. The collagen volume fraction (CVF%) of AF group was significantly higher than SR group. *P < 0.001. **d** Univariate linear regression curve of YKL-40 (*CHI3L1*) mRNA expression (independent variable) and CVF% (dependent variable)

**Fig. 4:**
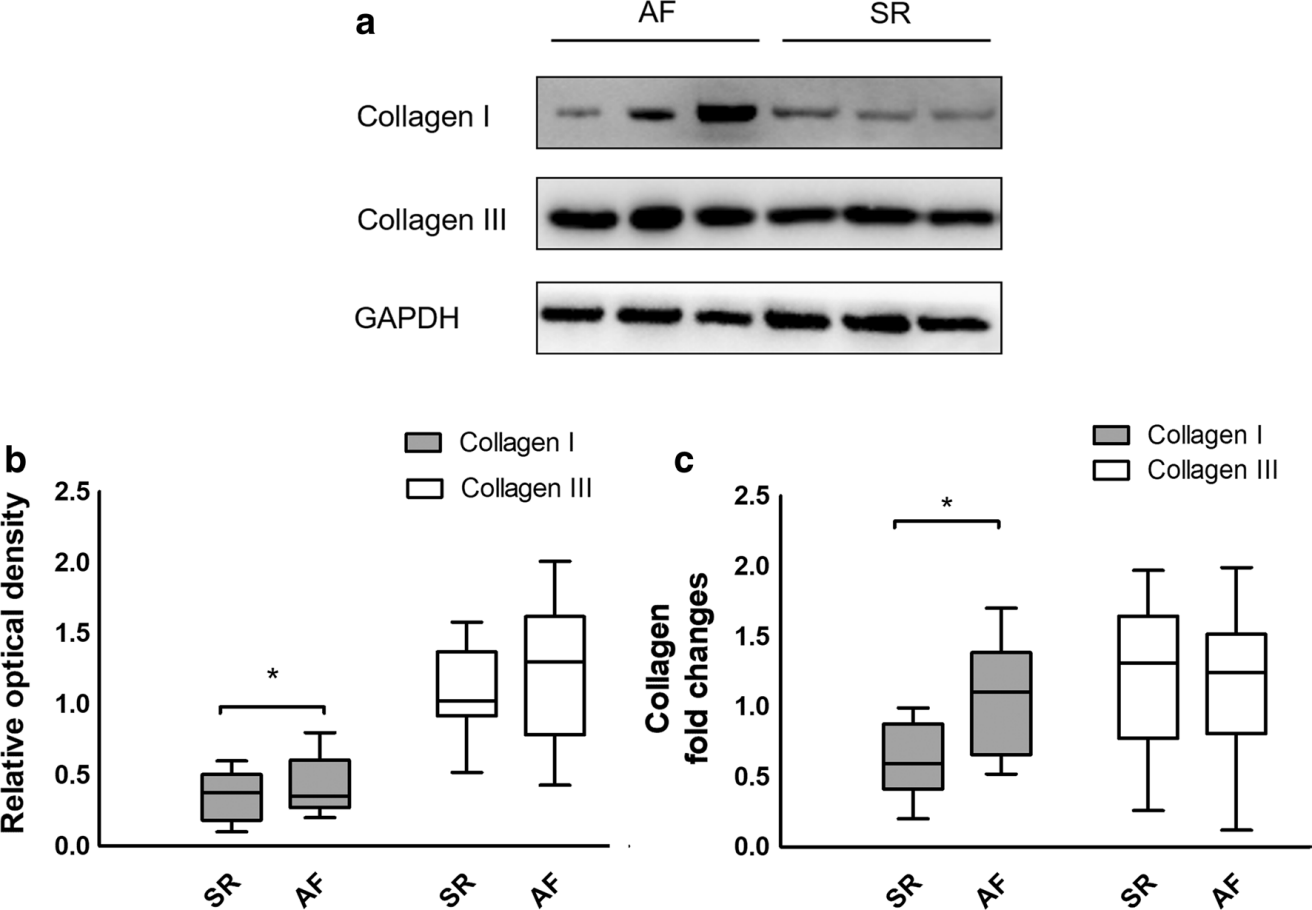
Western blotting and quantitative real-time PCR results of collagen I (*COL1A1*) and collagen III (*COL3A1*) expression in the atrial myocardium of patients with atrial fibrillation (AF, n = 28) and sinus rhythm (SR, n = 36). **a** Western blotting results of AF patients and SR patients (representative samples). **b** Relative optical density of western blotting results in the AF and SR groups. Relative optical density of Collagen I in AF group was significantly higher than SR group. **c** Quantitative real-time PCR results from the AF and SR groups. Collagen I mRNA expression of AF group was significantly higher than SR group. *P < 0.001

The Masson-stained sections of atrial myocardium are shown in Fig. 3a and b. The representative sections indicated more fibrotic and disordered myocardia in the AF group than in the SR group. As shown in Fig. 3c, the quantitative CVF% of the AF group was significantly higher than that of the SR group (40.87 ± 4.67 vs 31.74 ± 5.11, t = 7.357, *P *< 0.001). The western blotting and quantitative real-time PCR results conformed with the Masson staining, which indicated significantly higher collagen I mRNA and protein expression in the AF group (mRNA: 1.06 ± 0.39 vs 0.62 ± 0.24, t = 5.528, *P *< 0.001; protein: 0.44 ± 0.19 vs 0.34 ± 0.16, t = 2.109, *P *= 0.039), whereas there were no significant differences in collagen III expression between the 2 groups (mRNA: 1.11 ± 0.54 vs 1.24 ± 0.47, t = 1.048, *P *= 0.299; protein: 1.27 ± 0.47 vs 1.08 ± 0.30, t = 1.915, *P *= 0.060), as shown in Fig. 4. Univariate linear regression revealed a significant correlation between YKL-40 mRNA expression and CVF% (y = 3.576x + 26.205, *P *< 0.001), as shown in Fig. 3d.

### Multivariate linear regression analysis

Table 2 shows the results of the multivariate linear regression analysis for YKL-40 expression in EAT. Of the baseline characteristic enrolled in this model, only BMI was significantly associated with IOD of EAT in all patients (β = 0.328, *P *= 0.011).

**Table 2:** Multivariate linear regression analysis of the IOD of YKL-40 and baseline characteristics

Variables	B	SE	β	T	*P*
Constant	625.609	8256.052	–	0.076	0.940
Gender	2082.884	1565.724	0.200	1.330	0.189
Age	− 44.122	99.980	− 0.072	− 0.441	0.661
Smoking	− 844.645	1588.415	− 0.074	− 0.532	0.597
BMI	519.585	196.793	0.328	2.640	0.011
NYHA	− 543.476	914.067	− 0.091	− 0.595	0.555
T2DM	1257.607	1471.844	0.113	0.854	0.397
Hypertension	− 1607.303	1718.569	− 0.135	− 0.935	0.354
COPD	2694.578	1988.461	0.173	1.355	0.181
Stroke	4346.518	2416.941	0.245	1.798	0.078

*BMI* body mass index, *T2DM* type 2 diabetes mellitus

## Discussion

Atrial fibrillation, due to its high prevalence and severe complications of stroke and heart failure, remains a major threat to public health, with only limited and inconclusive evidence for its onset and development [9]. Atrial fibrosis is related to the structural remodeling of the atrium, which lays the basis for persistent and permanent AF [10]. EAT, or pericardial adipose tissue, previously thought to play a protective and metabolic role for the cardiovascular system, has been found to be highly correlated with the incidence and recurrence of AF in recent epidemiologic studies [11, 12]. The association between EAT and AF remains poorly understood; however, current evidence indicates that the arrhythmogenic mechanisms of EAT may include adipocyte infiltration, pro-fibrotic and pro-inflammatory paracrine effects, and oxidative stress [13]. We found that YKL-40 is more highly expressed in EAT than in other types of adipose tissue in AF patients, which may contribute to the development of AF.

YKL-40 plays a role in the activation of the innate immune system, extracellular matrix remodeling, and the differentiation and maturation of macrophages [14]. Several epidemiologic studies focused on the relationship between serum YKL-40 and AF, and showed that AF patients have higher levels of serum YKL-40, which is related to disease severity [15, 16]. However, it cannot be used as a biomarker for predicting the results of electrical cardioversion [16]. We found that YKL-40 mRNA levels were higher in the EAT of AF patients, while there was no significant difference in serum levels between the AF and SR groups, which suggested that EAT may be a depot for YKL-40 and affect the atrium by paracrine secretion. The difference between our results and previous studies in serum YKL-40 level can be explained in two major reasons. On one hand, Marott [15] led a cohort study focusing on the serum YKL-40 and the risk of developing AF, while we tested it on patients with existed AF. Also, our patients were all complicated by CAD, which was reported to be associated with YKL-40 level [17]. On the other hand, there was indeed a higher level of YKL-40 for AF patients, but the difference is not statistical significant, and the reason might be attributed to the relatively smaller sample size than previous study [16]. Although adipocytes may not express YKL-40, the macrophages and other inflammatory cells in the EAT can express it, as confirmed by the study of Catalan et al., who revealed higher expression of YKL-40 in the visceral adipose tissue of obese patients.

YKL-40, acting as a key factor in fibroblast proliferation and matrix deposition, is related to organic fibrosis, including lung [18] and hepatic fibrosis [19]. Based on our previous findings and the role played by atrial fibrosis in AF, we hypothesized that YKL-40 may induce AF by promoting atrial fibrosis. Hence, we determined the fibrosis level in all patients and analyzed its relationship with YKL-40 expression. We found that atrial fibrosis is prominent in AF patients compared with SR patients, and the quantitative index of CVF% has a linear relationship with YKL-40 mRNA levels. We also assessed the expression of representative collagen I and collagen III in all atrium samples, and noted a marked reduction in collagen I expression in the atrium, with no difference in collagen III. Similarly, Iwata et al. [20] found that YKL-40 secreted from macrophages in adipose tissue inhibits type I collagen degradation.

Furthermore, we tried to find possible risk factors affecting the expression of YKL-40 in EAT, and found that BMI is an independent risk factor. Epidemiologic studies and clinical data both indicate that obesity is commonly associated with AF, and stable weight loss decreases AF burden and AF recurrence following treatment [21]. Mild inflammation, insulin resistance, and structural remodeling in obese patients are believed to be the major reasons for the development of AF [22]. Another study found that serum YKL-40 is elevated in morbidly obese patients and declines after weight loss [23]. However, Nielsen et al. demonstrated that YKL-40 is independent of BMI as a biomarker for type 2 diabetes mellitus. Our study also found that the serum YKL-40 level did not differ between the AF and SR groups. The similarity between the two groups may be attributable to the limited sample size, but the expression in the EAT varies greatly between the groups.

Four aspects of the limitations of this study should be noted. Firstly, although significant differences have been identified, the relatively inadequate sample size is a disadvantage and the findings require evidence from further clinical and basic science studies. Secondly, all of the patients enrolled have coronary artery disease, which may affect the results. Thirdly, the study lacks healthy controls because healthy samples of EAT cannot be obtained for ethical reasons. Lastly, despite similar baseline characteristics, the results can still be influenced by mixed clinical factors. Notably, AF develops in multifaceted ways and is affected by numerous factors; the results in this study only provide some evidences that YKL-40 might be a booster to atrial fibrosis and AF, which require more experimental studies to validate.

## Conclusions

In summary, our study demonstrates that YKL-40 is highly expressed in EAT, especially in AF patients. YKL-40 expression in EAT is closely associated with atrial fibrosis, which is more marked in AF patients. We also found that BMI is the only risk factor for the expression of YKL-40 in EAT. Future studies should focus on the cause–effect relationship of YKL-40 and AF, and basic research should address how YKL-40 activates fibrotic processes in the atrium, which may provide novel targets for the prevention and treatment of AF.

## Abbreviations

AF: atrial fibrillation
SR: sinus rhythm
EAT: epicardial adipose tissue
SAT: subcutaneous adipose tissue
PAT: paracardial adipose tissue
CVF%: volume fraction of collagen
IOD: integrated optical density
BMI: body mass index
LVEF: left ventricular ejection fraction
LAD: left atrial diameter
T2DM: type 2 diabetes mellitus
COPD: chronic obstructive pulmonary disease
NYHA: New York Heart Association
